# Multifocal Dural Arteriovenous Fistula Formation Following Successful Endovascular Treatment of a Traumatic Direct Carotid Cavernous Fistula

**DOI:** 10.7759/cureus.96254

**Published:** 2025-11-06

**Authors:** Fumitaka Koga, Atsushi Ogata, Kenji Suzuyama, Jun Masuoka, Tatsuya Abe

**Affiliations:** 1 Department of Neurosurgery, Faculty of Medicine, Saga University, Saga, JPN; 2 Department of Neurosurgery, Karatsu Red Cross Hospital, Karatsu, JPN

**Keywords:** carotid cavernous fistula, dural arteriovenous fistula, endovascular treatment, multifocal, trauma

## Abstract

The development of dural arteriovenous fistulas (DAVFs) following treatment of a direct carotid-cavernous fistula (CCF) is extremely rare. We report the first case of multifocal DAVF formation at distinct anatomical sites after direct CCF treatment. A 76-year-old woman underwent successful endovascular therapy for a traumatic right-sided direct CCF. One month post-embolization, angiography revealed DAVFs at the left cavernous sinus and right inferior petrosal sinus. The left cavernous sinus DAVF was treated with transvenous embolization, while the asymptomatic right inferior petrosal sinus DAVF underwent spontaneous regression. Clinicians should maintain long-term vigilance following direct CCF treatment, as multiple DAVFs may develop at distant anatomical sites.

## Introduction

Direct carotid-cavernous fistulas (CCFs) are high-flow arteriovenous shunts between the cavernous segment of the internal carotid artery and the cavernous sinus, typically resulting from trauma or rupture of intracavernous carotid aneurysms [1,2]. These lesions are classified as Barrow type A and require prompt endovascular treatment due to their associated morbidity and low rate of spontaneous closure [1,3].

Dural arteriovenous fistulas (DAVFs) are acquired arteriovenous shunts between dural arteries and venous structures. Unlike direct CCFs, DAVFs are typically low-flow lesions, although those with cortical venous reflux can cause serious neurological complications requiring intervention.

The development of DAVFs following treatment of direct CCFs is extremely rare but clinically important, as they may mimic recurrence of the original direct CCF and may require additional treatment depending on their venous drainage pattern. Previous case reports have described DAVFs developing months after successful occlusion of direct CCFs [4-8].

While several isolated cases of DAVFs appearing after direct CCF treatment have been documented, most involve a single DAVF at the cavernous sinus [4,6,7]. Only one case of bilateral cavernous sinus DAVFs has been reported in the literature [5]. The development of multiple DAVFs at different anatomical locations following direct CCF treatment has not been previously reported.

We report a case of traumatic direct CCF in which DAVFs developed at the contralateral cavernous sinus and ipsilateral inferior petrosal sinus (IPS) following endovascular treatment, representing the first case of multifocal DAVF formation at distinct anatomical sites after direct CCF treatment.

## Case presentation

**Figure 1:**
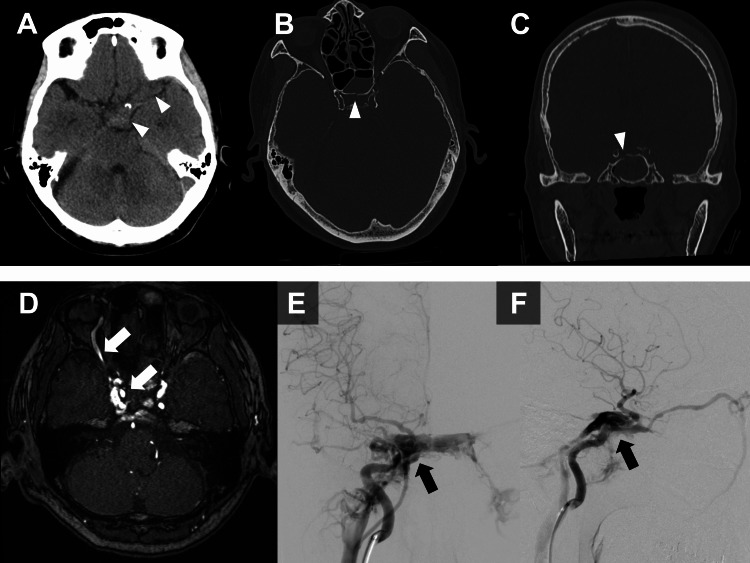
Initial imaging of a traumatic direct carotid-cavernous fistula. (A) Axial unenhanced computed tomography (CT) showing subtle hyperdensity in the suprasellar cistern (arrowheads), consistent with traumatic subarachnoid hemorrhage. (B) Axial CT (bone window) demonstrating a sphenoid bone fracture (arrowhead). (C) Coronal CT (bone window) demonstrating a sphenoid bone fracture (arrowhead). (D) Axial magnetic resonance angiography (MRA) source image on day 9 of hospitalization showing an arteriovenous shunt in the cavernous sinus region with a dilated right superior ophthalmic vein (white arrows). (E) Anteroposterior right internal carotid artery (ICA) angiography confirming a direct carotid-cavernous fistula between the cavernous ICA and right cavernous sinus (black arrow). (F) Lateral right ICA angiography confirming a direct carotid-cavernous fistula (black arrow).

A 76-year-old woman was emergently transported to our hospital following a motor vehicle collision while riding a bicycle. On arrival, she presented with a mild disturbance of consciousness (Glasgow Coma Scale (GCS) score of 13). CT revealed traumatic subarachnoid hemorrhage (Figure 1A), left temporal bone and anterior skull base fractures (Figures 1B-1C), and a C3 vertebral body fracture. The patient was managed conservatively with observation.

On the 9th day of hospitalization, magnetic resonance angiography (MRA) was performed to screen for traumatic intracranial aneurysms. It incidentally demonstrated a suspected arteriovenous shunt in the cavernous sinus region (Figure 1D). Cerebral angiography was performed on the 14th day of hospitalization, confirming the diagnosis of a right-sided direct CCF (Figures 1E-1F). A balloon test occlusion of the internal carotid artery revealed intolerance to vessel occlusion with symptom development. At this time point, no DAVFs were identified on angiography.

**Figure 2:**
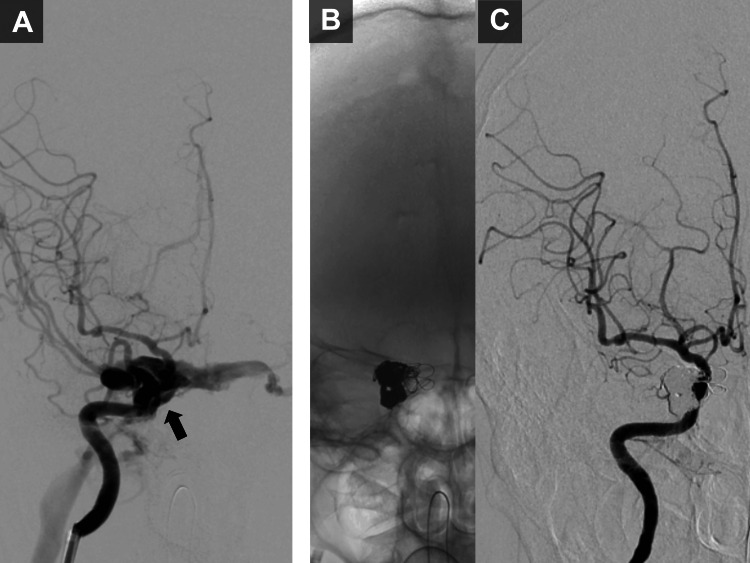
Endovascular treatment of a direct carotid-cavernous fistula. (A) Anteroposterior right internal carotid artery (ICA) angiography demonstrating a high-flow direct carotid-cavernous fistula (CCF) (black arrow). (B) Anteroposterior radiograph after embolization showing coils within the right cavernous sinus. (C) Anteroposterior right ICA angiography after embolization demonstrating complete occlusion of the CCF.

On the 45th day post-injury, the patient developed right-sided proptosis, conjunctival injection, and diplopia. Two months post-injury, endovascular embolization was performed. A microcatheter was advanced transarterially to the fistulous point. To prevent coil migration into the right internal carotid artery, a microballoon catheter was positioned in the right internal carotid artery. Transvenously, a microcatheter was navigated to the right cavernous sinus via the right inferior petrosal sinus (IPS). With intermittent microballoon inflation, detachable coils were deployed through both microcatheters to occlude the right cavernous sinus, successfully closing the fistula (Figures 2A-2C). Following embolization, the patient’s symptoms of proptosis, conjunctival injection, and diplopia showed partial improvement but persisted. Again, no DAVFs were observed at this stage.

**Figure 3:**
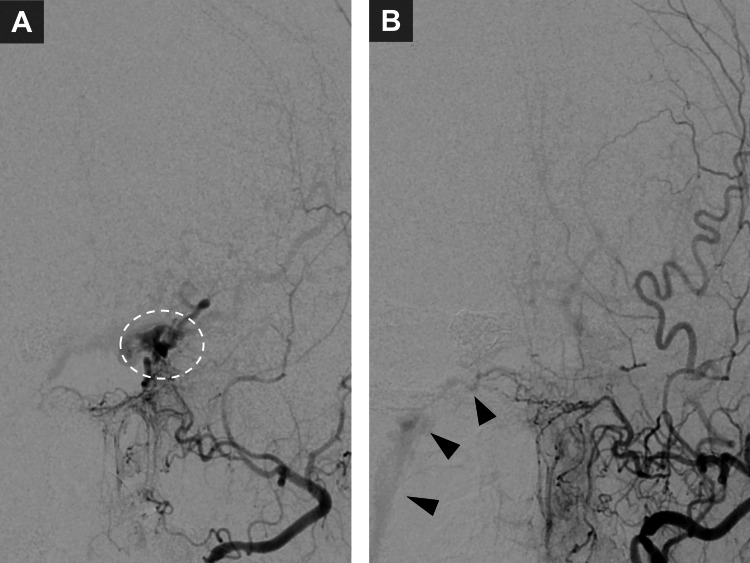
Delayed dural arteriovenous fistulas (DAVFs) at one-month follow-up. (A) Anteroposterior left external carotid artery (ECA) angiography one month after embolization showing a dural arteriovenous fistula (DAVF) at the left cavernous sinus (dotted circle). (B) Anteroposterior left ECA angiography after transvenous embolization demonstrating occlusion of the left cavernous sinus DAVF. Also shown is a separate DAVF at the right inferior petrosal sinus (IPS).

At three months post-injury (one month after the initial embolization), MRA suggested possible recurrence, prompting a planned additional embolization procedure. At this stage, the ocular symptoms persisted. Cerebral angiography revealed no recurrence of the direct CCF; however, DAVFs were now evident at the left cavernous sinus and right IPS (Figure 3A). These DAVFs were not visualized on bilateral internal carotid artery angiography. The left cavernous sinus DAVF demonstrated cortical venous reflux and underwent transvenous embolization. A microcatheter was advanced transvenously to the left cavernous sinus via the left IPS, and detachable coils were deployed to occlude the left cavernous sinus, successfully eliminating the fistula (Figure 3B).

**Figure 4:**
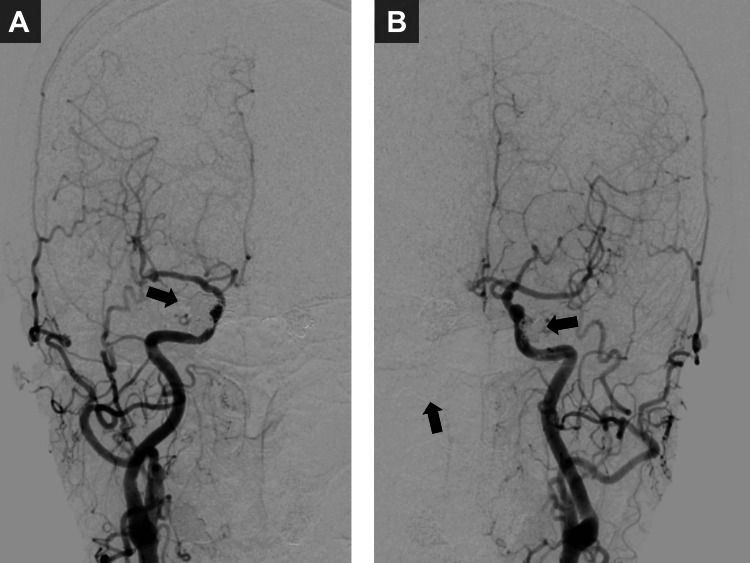
Complete obliteration at final follow-up. (A) Anteroposterior right common carotid artery (CCA) angiography at follow-up demonstrating spontaneous regression of the right inferior petrosal sinus (IPS) dural arteriovenous fistula (DAVF) with complete obliteration of all arteriovenous shunts. (B) Anteroposterior left internal carotid artery (ICA) angiography after transvenous coil embolization confirming complete obliteration of the left cavernous sinus DAVF.

The right IPS DAVF remained asymptomatic without cortical venous reflux and was managed conservatively. Follow-up angiography at three months after the second embolization (six months post-injury) demonstrated spontaneous regression of this lesion (Figure 4). The patient’s right-sided proptosis, conjunctival injection, and diplopia showed marked improvement at six months post-injury.

## Discussion

This case demonstrates the rare occurrence of multiple DAVFs developing after successful treatment of a traumatic direct CCF. Based on reported cases in the literature [4-7], the time from injury to initial treatment varies considerably, ranging from 2 weeks to 15 months (Table 1). In our case, although the direct CCF was detected on MRA 9 days after injury, the patient remained asymptomatic and was managed conservatively. Treatment was initiated 2 months after injury when symptoms developed, which falls within the range of previously reported cases.

**Table 1:** Summary of reported cases with DAVF development following direct CCF treatment. CCF: Carotid-cavernous fistula; CS: Cavernous sinus; DAVF: Dural arteriovenous fistula; EC-IC: External carotid-internal carotid; ICA: Internal carotid artery; IPS: Inferior petrosal sinus; TAE: Transarterial embolization; TVE: Transvenous embolization.

Author (Year)	Time from injury to initial treatment	Initial treatment	Time from initial treatment to DAVF development	Location of DAVF	Follow-up duration after DAVF treatment
Terada T et al. (2002) [4]	6 months	Detachable balloon	5 months	Right CS	Not reported
Lv XL et al. (2008) [5]	15 months	Covered stent	9 months	Bilateral CS	14 months
Gao BL et al. (2009) [6]	1 month	Detachable balloon (bilateral)	7 months	Left CS	3 months
Yoshino H et al. (2011) [7]	2 weeks	ICA trapping + EC-IC bypass	10 days	Right CS	Not reported
Present case (2025)	2 months	Balloon-assisted TAE and TVE	1 month	Left CS and right IPS	6 months

The pathogenesis of DAVFs following direct CCF treatment is multifactorial. Venous hypertension within the cavernous sinus system is considered a primary contributing factor [9,10]. Prolonged elevation of venous pressure throughout the cavernous sinus network likely initiates the cascade leading to DAVF formation. Sinus thrombosis following embolization represents another crucial mechanism [4], as the placement of embolic material within the cavernous sinus leads to local thrombosis and subsequent inflammatory changes. This process may trigger angiogenic factor expression, which has been implicated in DAVF development [11,12].

The timing of DAVF development after initial treatment also varies among reported cases [4-7], ranging from 10 days to 9 months (Table 1). Notably, the case reported by Yoshino H et al. showed remarkably early development of a DAVF at 10 days post-treatment, which is significantly earlier than other reports [7]. This early onset can be attributed to the presence of a sphenoid bone fracture and ipsilateral middle meningeal artery extravasation observed on initial angiography, suggesting a different pathogenetic mechanism compared to other cases. Such findings were not observed in the present case. Therefore, DAVFs developing due to prolonged venous hypertension and subsequent cavernous sinus thrombosis and inflammation following initial embolization may be more likely to occur 3 to 9 months after direct CCF treatment.

Among the reported cases, only one previous case involved bilateral cavernous sinus DAVFs with mirror-image locations [5]. Our case is unique in that the DAVFs developed at different anatomical sites, the left cavernous sinus and right IPS. In our case, the initial high-flow direct CCF caused widespread venous hypertension not only in the ipsilateral cavernous sinus but also extended to the contralateral cavernous sinus via intercavernous connections and to the ipsilateral IPS through direct anatomical continuity [9,10]. This prolonged venous hypertension throughout the interconnected cavernous sinus network likely created multiple vulnerable sites for DAVF formation. Following embolization, the combination of preceding venous hypertension, local thrombosis, and inflammatory changes [4,11,12] may have triggered DAVF development at these multiple anatomically connected but distinct locations. This demonstrates that DAVFs can occur at sites distant from the original lesion, extending beyond the typical ipsilateral cavernous sinus involvement.

All previously reported cases of secondary DAVFs following direct CCF treatment underwent embolization. However, in our case, the right IPS DAVF showed antegrade venous drainage without symptoms such as tinnitus and was managed conservatively. This lesion subsequently underwent spontaneous regression. While spontaneous occlusion of DAVFs is rare, it can occur through several mechanisms. DAVFs are often associated with venous sinus thrombosis, which can be both a cause and a consequence of the fistula [4,9,10]. Progressive thrombosis within the draining venous sinus can lead to spontaneous closure by obliterating venous outflow [13,14]. Contrast agents used during diagnostic angiography may also contribute to spontaneous regression through their effects on endothelial function and local hemodynamics, potentially promoting thrombosis within low-flow DAVFs [13,14]. In our case, the antegrade venous drainage pattern suggesting low-flow physiology and repeated angiographic examinations may have facilitated spontaneous regression of the right IPS DAVF.

## Conclusions

DAVFs can develop as a delayed sequela following the treatment of direct CCFs. Careful long-term follow-up is essential, even after successful closure of a direct CCF. Clinicians should remain aware that multiple DAVFs may arise at sites distant from the original lesion. The management of these delayed-onset DAVFs should follow the same principles applied to conventional DAVFs.
